# Surface display as a functional screening platform for detecting enzymes active on PET

**DOI:** 10.1186/s12934-021-01582-7

**Published:** 2021-05-01

**Authors:** Sophia A. H. Heyde, Jenny Arnling Bååth, Peter Westh, Morten H. H. Nørholm, Kenneth Jensen

**Affiliations:** 1 Novozymes A/S, Biologiens Vej 2, 2800 Kgs. Lyngby, Denmark; 2 Novo Nordisk Foundation Center for Biosustainability, Technical University of Denmark, Kemitorvet B220, 2800 Kgs. Lyngby, Denmark; 3 Department of Biotechnology and Biomedicine, Technical University of Denmark, Søltofts Plads, 2800 Kgs. Lyngby, Denmark

**Keywords:** PETase, *Ideonella sakaiensis*, Surface display, Extracellular protein production, *E. coli*

## Abstract

Poly(ethylene terephthalate) (PET) is the world’s most abundant polyester plastic, and its ongoing accumulation in nature is causing a global environmental problem. Currently, the main recycling processes utilize thermomechanical or chemical means, resulting in the deterioration of the mechanical properties of PET. Consequently, polluting *de novo* synthesis remains preferred, creating the need for more efficient and bio-sustainable ways to hydrolyze the polymer. Recently, a PETase enzyme from the bacterium *Ideonella sakaiensi*s was shown to facilitate PET biodegradation, albeit at slow rate. Engineering of more efficient PETases is required for industrial relevance, but progress is currently hampered by the dependency on intracellular expression in *Escherichia coli*. To create a more efficient screening platform in *E. coli*, we explore different surface display anchors for fast and easy assaying of PETase activity. We show that PETases can be functionally displayed on the bacterial cell surface, enabling screening of enzyme activity on PET microparticles – both while anchored to the cell and following solubilization of the enzymes.

**Supplementary Information:**

The online version contains supplementary material available at 10.1186/s12934-021-01582-7.

## Introduction

Poly(ethylene terephthalate) (PET) is one of the most commonly produced plastics (with an annual manufacturing of over 30 million tons), appreciated for its low cost, robustness, and high durability. This highly inert polyester consists of repeating units of the aromatic building block terephthalate (TPA) linked to ethylene glycol (EG) [1]. The inherent non-degradable properties that make PET an excellent material, unfortunately have the huge environmental drawback of substantial accumulation in landfills and in the ecosystem [2]. Increased build-up of plastic waste together with the fact that PET is produced from fossil feedstock call for efficient recycling and remediation strategies. These are currently dominated by thermo-mechanical and chemical means, with drawbacks like downcycling and the use of hazardous chemicals [3]. An environmentally friendly and less destructive recycling method is biodegradation, and recently, the interest in enzymatic PET degradation has increased tremendously [4].

The crystallinity and the limited accessibility to the ester linkages make PET unsusceptible to enzymatic hydrolysis, but a number of ester-cleaving enzymes have been reported active on the polyester [1]. The most promising candidates are classified as cutinases (EC 3.1.1.74), but the turnover rates are only low to moderate, reflecting that PET is an unnatural substrate for these enzymes [5]. A breakthrough in the field was the discovery of *Ideonella sakaiensis* in 2016, a bacterium able to utilize PET as its sole carbon source by converting it into TPA and EG, which was further metabolized. The initial catabolic pathway by *I. sakaiensis* involves two novel enzymes. The initial hydrolysis step of PET is facilitated by an extracellular enzyme homologous to cutinases, but with an extraordinary capability to degrade PET, hence classified as a PETase (EC 3.1.1.101). The hydrolysis products, of which Mono-(2-hydroxyethyl)terephthalic acid (MHET) comprises a major part, are transported into the periplasmic space, and degraded to TPA and EG by the second novel enzyme, classified as a MHETase (EC 3.1.1.102) [6].

However, even with the PETase’s promising catalytic properties, problems with expression, purification and stability make it inadequate for industrial purposes [7, 8]. Several engineering efforts have resulted in a few catalytically improved and more thermostable variants [7, 9–12]. Examples of improved variants of the *I. sakaiensis* PETase are the *Is*PETase^Austin^ [9], and *Is*PETase^Son^ [7]. The improvements in activity towards PET were achieved using two different strategies. In *Is*PETase^Austin^, the focus was to narrow the active site and to make it more cutinase-like by introducing the double mutation S238F and W159H, whereas the triple mutation S121E, D186H and R280A in *Is*PETase^Son^ was centred around stabilizing loop regions, thereby improving the overall temperature stability. Irrespectively of the strategy, both variants were shown to clearly outcompete the wildtype PETase (*Is*PETase^WT^).

Protein engineering efforts have all been done in *E. coli*, despite the complications imposed by the need for the release of intracellularly produced enzyme variants. Interestingly, only 2–3 mutations have been incorporated in the *Is*PETase^Austin^, and *Is*PETase^Son^ variants. One could speculate if use of a more amenable system, circumventing the limitations imposed by intracellular localization, would enable a more efficient protein engineering workflow for improving the *Is*PETase. As there is still no report of the PETase being successfully upscaled in an industrially relevant microbe, *E. coli* has remained the organism of choice, able to produce small amounts of the enzyme, albeit most of it ends up in inclusion bodies [8].

For large-scale production and screening of metagenomic samples for novel enzymes active on PET, there is a need for a robust and simple expression procedure. Bacterial surface display of enzymatically active proteins is a promising strategy to engineer whole-cell catalysts, but also to overcome the bottleneck of inefficient enzyme secretion and cytoplasmatic toxicity, enabling simplification of production procedures as well as downstream processes. Since the first attempts in the late '80s , surface display is steadily gaining popularity and has been explored in most industrially relevant microbial platforms. Nonetheless, *E. coli* remains the most frequently used organism for surface display with a great variety of membrane anchors available [13]. Herein, we present a method for surface display expression of PETase variants using *E. coli* protein production strain BL21(DE3). This expression strategy allows for quick screening of the produced variants, where the lysis step and the formation of inclusion bodies are avoided. The procedure provides a convenient screening platform that could be applied to PETase variants in an industrially relevant organism suitable for integration into high throughput screening systems.

## Methods

### Construction of expression vectors

Genes encoding the three *Is*PETase variants (*Is*PETase^WT^, *Is*PETase^Austin^, *Is*PETase^Son^) [7, 9, 11] were synthesized according to the published codon-optimized sequence from Austin et al., 2018 [9] (final sequences can be found in Additional file 1: Sequence list) and cloned scarlessly into vectors pKSD:LppOmpA-NB and pKSD:NB-C-IgAP [14] using uracil-excision (USER) based cloning [15] and primers 1–7 (Additional file 1: Table S1) without their leader sequence and Met codon present. Sequences encoding TEV cleavage sites (ENLYFQ/G) were added in frame to either both sites of the enzyme sequence (IgAP module, STOP codon removed) or upstream of the N-terminus only (LppOmpA module, STOP codon intact) to enable cleavage from the surface anchor. We additionally added hexa-histidine tags C-terminally prior to the terminal TEV cleavage site (C-IgAP module) or STOP codon (LppOmpA module) for purification of the different *Is*PETase variants after TEV cleavage. Both the TEV cleavage sites and hexa-histidine tags were fused directly to the coding sequence without use of linker sequences.

### Expression of surface-displayed *Is*PETase variants

All pKSD expression vectors were transformed into BL21(DE3) competent *E. coli* (NEB) via heat shock and according to the manufacturer’s description. Expression cultures were cultivated at 37 °C in 500 ml Erlenmeyer shake flasks without baffles in a culture volume of 50 mL lysogenic broth medium supplemented with 50 µg/mL Kanamycin. Cultures were induced at an optical density of 0.3–0.5 at 600 nm via the addition of l-rhamnose (5 mM final concentration) and subsequently incubated for 20 h at 30 °C or 60 h at 16 °C (as stated) shaking at 250 rpm.

### GFP binding assay to assess surface display efficiency

The nanobody:GFP binding assay was performed as previously described [14]. Surface display expression was terminated via centrifugation for 4 min at 2300*g* and cells were resuspended in 50 mM Tris buffer (pH 7.5). Purified GFP (for detailed purification protocol, see [14]) was added to a final concentration of 0.06 mg/mL and binding was allowed for 20 min at 30 °C while shaking at 250–300 rpm. Post GFP binding, cells were washed twice in 50 mM Tris buffer and 50 µL of washed cell suspension was mixed with 100 µL 50 mM Tris buffer and transferred into a 96-well microtiter plate (Sigma-Aldrich). Optical density at 630 nm and GFP fluorescence (excitation: 485 nm, emission: 528 nm) were measured in a SynergyH1 plate reader (BioTek) in transparent or opaque microtiter plates (Sigma-Aldrich), respectively.

### TEV cleavage

For TEV cleavage of the surface-displayed *Is*PETase variants, cells were harvested for 5 min at 4000 g, washed twice in 10 mM Tris-HCl (pH 7.5) and subsequently resuspended in the same buffer (100 µL per ODU). Commercial TEV protease (Sigma-Aldrich) was added to the cell suspension resulting in a final concentration of 1 Unit/ODU and the reaction incubated overnight at 4 °C, rotating at 10 rpm on a Blood Mixer Intelli-Mixer RM-2 S. Post incubation, cells were pelleted at 5000 g for 10 min and the supernatant was collected for activity analysis on para-nitrophenol (*p*NP)-acetate/butyrate and PET, and SDS-PAGE. Solubilized *Is*PETase variants were purified using His GraviTrap TALON (GE Healthcare Life Sciences) columns according to the manufacturer’s instructions. In short, the supernatant was applied to a Ni–NTA agarose column. After washing with 20 mM sodium phosphate buffer containing 500 mM NaCl (buffer A) and 30 mM imidazole, the bound proteins were eluted with 300 mM imidazole in buffer A. Finally, the imidazole was removed using a PD-10 column (Amersham biosciences) equilibrated with 50 mM HEPES (pH 8) buffer. The *Is*PETase^Austin^ purified control was purified as described in [16].

### *p* NP-acetate/butyrate activity assay

20 µL whole cells (OD_630_ = 0.1) displaying *Is*PETase variants on the cell surface, supernatant of washed cells after TEV cleavage, and purified *Is*PETase (0.1 µg/mL) were incubated with 1 mM *p*NP-acetate or *p*NP-butyrate (Sigma-Aldrich) in 50 mM phosphate buffer (pH 7) for 5 min at 24 °C. The release of *p*NP was monitored at a wavelength of 405 nm in a SynergyH1 plate reader (BioTek). For *p*NP-acetate assays on whole cells, *E. coli* BL21(DE3) cells not harbouring any of the surface-display expression vectors were used as negative control (NC). Since His-tag purified *Is*PETases were stored in 50 mM HEPES buffer (pH 8), the same buffer was respectively used as negative control for the *p*NP-acetate assay on purified enzymes.

### Protein gels and western blot

For in-gel fluorescence, cells were resuspended after nanobody:GFP binding to a concentration of 0.05 ODU/µL and 20 µL were mixed with 10 µL 2×Laemmli sample buffer and 0.5 µL benzonase nuclease (≥ 250 units/µL, Sigma). 15 µL of the whole sample were loaded onto a 4–20% Criterion™ TGX™ gel (Bio-Rad) at non-denaturing conditions and run for 30 min at 200 V. Fluorescent protein bands were visualized using UV light and a GelDoc XR + imaging system (Bio-rad). Total protein was assessed afterwards by staining with Novex SimplyBlue SafeStain. For *Is*PETase detection by western blot, samples were mixed with Laemmli sample buffer as described above, boiled for 5 min and separated via SDS-PAGE (4–20% Criterion™ TGX™ gel (Bio-Rad) at denaturing conditions). Total protein was transferred onto a nitro cellulose membrane using the iBlot™ 1 gel transfer system (Invitrogen). Blots were blocked with TBS-1% dried skim milk powder and developed with sequential incubations of mouse anti penta histidine tag (α-His) antibody (Bio-Rad, MCA5995P) and horse radish peroxidase (HRP) conjugated secondary goat anti mouse IgG (Sigma-Aldrich, M8642-1MG).

### Activity measurement on suspended PET

Activity measurements of whole cell suspension (OD_630_ = 0.1) surface-displaying PETase variants and free PETase enzyme (post TEV cleavage, corresponding roughly to a concentration of 0.01–0.1 µM) on a suspended, semi-crystalline PET powder (Goodfellow Co, ES306031) were executed by adapting a plate reader-based assay previously described [17]. In short, reactions were performed in sealed low binding 96-well plates (Greiner Bio-One™ 655900), incubated at 40 °C, in 50 mM sodium phosphate pH 8, at 1100 rpm in an Eppendorf thermomixer for 4 h. PET concentration was 15 g/L and enzyme sample volume was 50 µL in a total volume of 250 µL. Reactions were quenched by centrifugation, the supernatant withdrawn, and formation of soluble products was detected by measuring the absorbance at 240 nm.

## Results and discussion

### Selection of membrane anchors for *Is*PETase surface display in *E. coli*

To overcome the bottleneck of inefficient heterologous expression and secretion of *Is*PETase variants in substantial amounts for screening and characterization, we adapted a surface display system previously described [14] to enable activity screening of *Is*PETase variants without the need for secretion into the growth medium. The system developed by Wendel et al. consists of both N- and C-terminal transmembrane anchors fused to the protein of interest. Sandwiched in between the membrane anchor and the protein of interest is a single domain camelid-derived antibody fragment (nanobody; NB) with high affinity for GFP [18], binding the fluorophore in a stable complex, thus allowing visualization of the surface display module when exposed on the cell surface.

**Fig. 1:**
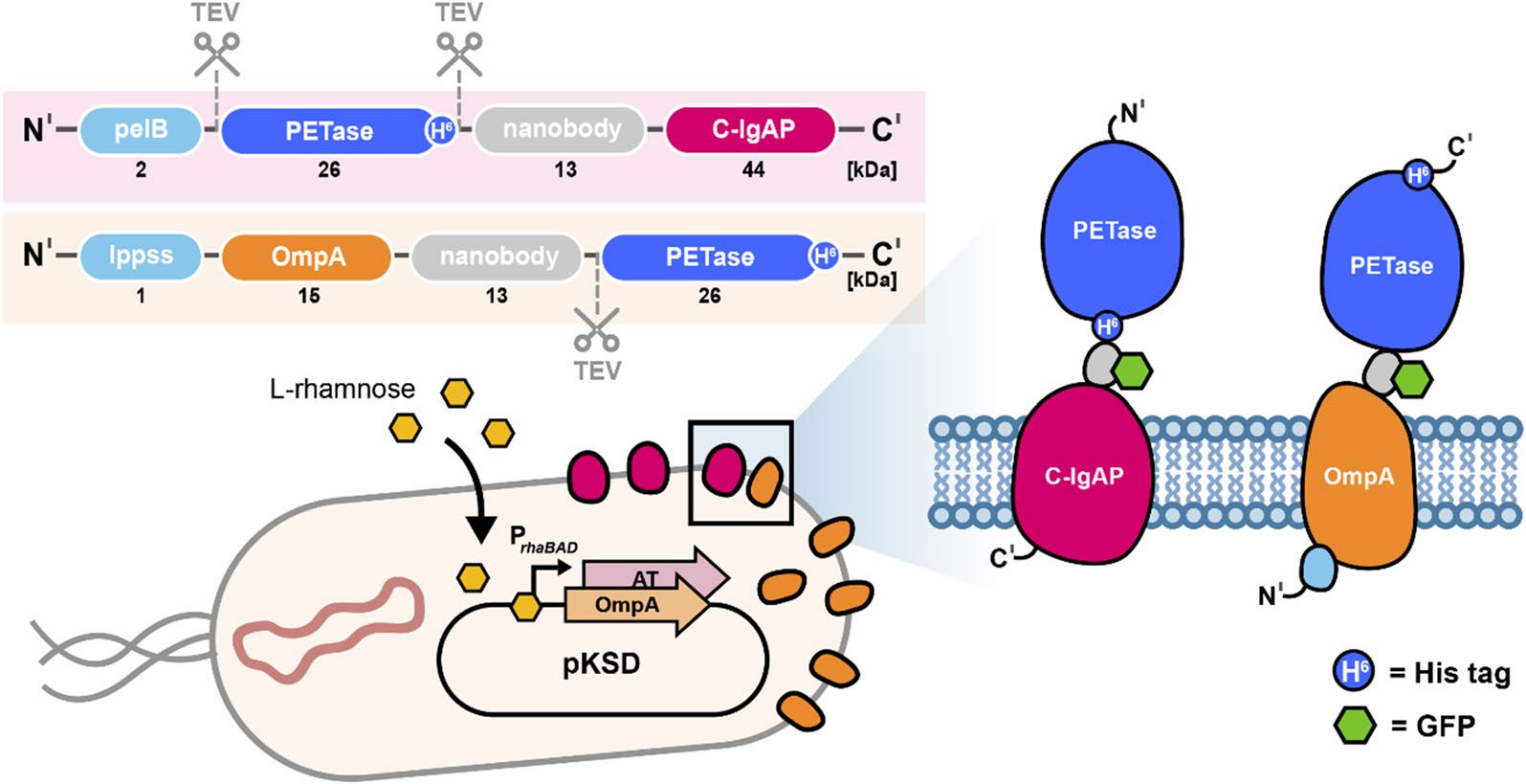
Schematic illustration of surface display modules for *Is*PETase expression. C-IgAP (purple) and LppOmpA (orange) anchor module and orientation of *Is*PETases within the modules are shown. TEV cleavage sites and His-tags (6x histidine) are indicated. N-terminal signal sequences (lppss and pelB) precede both constructs. A schematic illustrating both modules in the bacterial outer membrane is displayed (right side), indicating the location of nanobody:GFP (gray:green) binding. The pelB signal sequence is cleaved during translocation and therefore not displayed in the schematic

Two different membrane anchors were explored: (i) the C-terminal translocation unit of the *Neisseria gonorrhoeae* autotransporter IgA protease (C-IgAP) and (ii) a LppOmpA fusion protein consisting of the Lpp signal peptide followed by five transmembrane segments (residues 66 to 180) of *E. coli* outer membrane protein A (OmpA) (Fig. 1). All three versions of the *Is*PETase (*Is*PETase^WT^, *Is*PETase^Austin^, and *Is*PETase^Son^) were fused to the GFP-nanobody (part of the anchoring module) either C-terminally with a PelB signal peptide directly fused to the N-terminus of the enzyme, or N-terminally allowing a free C-terminus, resulting in a total module size of 85 or 55 kDa, respectively. The two modules insert in antagonal orientation into the bacterial cell membrane. This way, *Is*PETase variants are oriented in opposite directions and can be translocated across the membrane without direct fusion to the signal peptide in the N-terminal LppOmpA module. In both cases, protein production is under the control of the rhamnose-inducible rhaP_BAD_ promoter and successful display on the cell surface can be assessed upon GFP binding. This allows an easy and fast estimation of the relative surface display capacity. Furthermore, the introduction of TEV cleavage sites at both the N- and C-termini (C-IgAP module) or solely the N-terminus (LppOmpA module) along with a C-terminal histidine-tag prior to the TEV site allow the controlled release of the *Is*PETase variants into solution at any desired time point, as well as their subsequent purification.

**Fig. 2:**
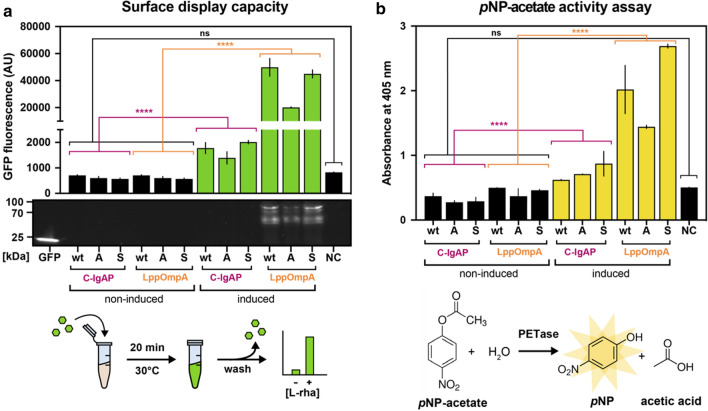
Functional surface display of *Is*PETase variants comparing a C-IgAP and LppOmpA anchor module. **a** Whole-cell and in-gel fluorescence for *Is*PETase variants (wt: wild type; A: Austin, S: Son) surface-displayed using either C-IgAP or LppOmpA module with and without L-rhamnose induction. The nanobody:GFP binding workflow is indicated. Unbound GFP was run on the outer left lane. Samples have been normalized to ODU. **b** Absorbance change at 405 nm measured on *p*NP-acetate for *Is*PETase variants expressed via the two modules. Active PETase degrades *p*NP-acetate to *p*NP, and acetic acid as illustrated. NC: Negative control BL21(DE3) cells not harboring the expression vector. Statistical evaluation: Multiple comparison analysis (two-way ANOVA); ns: no significant statistical difference, ****: p-value < 0.0001

The efficiency of the different transmembrane anchors for displaying the *Is*PETase was evaluated on whole cells. In short, this was done by isolating the cells post induction (expression at 30 °C), and quantifying the signal derived from the GFP bound to the surface-exposed nanobody. This has previously been shown to be an excellent proxy for measuring total surface displayed protein [14]. The LppOmpA anchor successfully displayed the *Is*PETase-containing surface display modules, as confirmed via fluorescence measurement in a microtiter plate reader as well as via in-gel fluorescence of OD-normalized whole-cell samples after incubation with GFP (Fig. 2a). At non-denaturing conditions, concise bands corresponding to a complex of the surface display module bound to GFP via the nanobody element [theoretical sizes 112 (C-IgAP) and 82 kDa (LppOmpA)] could be detected for the LppOmpA module, whereas no fluorescence appeared to originate from either the C-IgAP module or the unbound GFP (27 kDa). The additional bands on the native gel most likely stem from partially denatured and incompletely or incorrectly folded LppOmpA modules containing the nanobody-GFP complex. Induced cultures were highly fluorescent compared to non-induced ones, with cultures expressing the LppOmpA anchor module emitting significantly higher fluorescence than the C-IgAP cultures. This suggests that the N-terminal fusion construct is the most efficient for signal peptide-free *Is*PETase expression. Fusion of the pelB signal peptide directly onto the enzymes’ N-terminus (C-IgAP module) was previously observed to be possible, but inefficient for *Is*PETase secretion [8] in line with our observations. A further advantage of the LppOmpA anchor module is the free C-terminus on the PETase, which readily allows fusion of tags or reporter proteins for downstream processing.

To investigate whether *Is*PETase enzymes displayed on the bacterial cell surface were correctly folded in an active conformation, we screened the six candidate constructs for activity on *p*NP-acetate, a standard chromogenic model substrate for esterases [19]. For the surface display modules that were N-terminally fused onto the LppOmpA fusion protein, and in good correlation with the GFP binding assay, higher amounts of released *p*NP could be detected as a larger shift in absorbance (Fig. 2b). Identical results could be confirmed using the comparable compound *p*NP-butyrate as substrate (Additional file 1:  Figure S1). Despite the high background activity of the BL21(DE3) strain on the *p*NP-acetate, a significant activity increase for all the membrane-bound *Is*PETase variants post induction could be detected when compared to non-induced cultures. Based on these observations, the LppOmpA anchor module was chosen for all further experiments.

### Surface-bound *Is*PETase^WT^ is active on PET microparticles

**Fig. 3:**
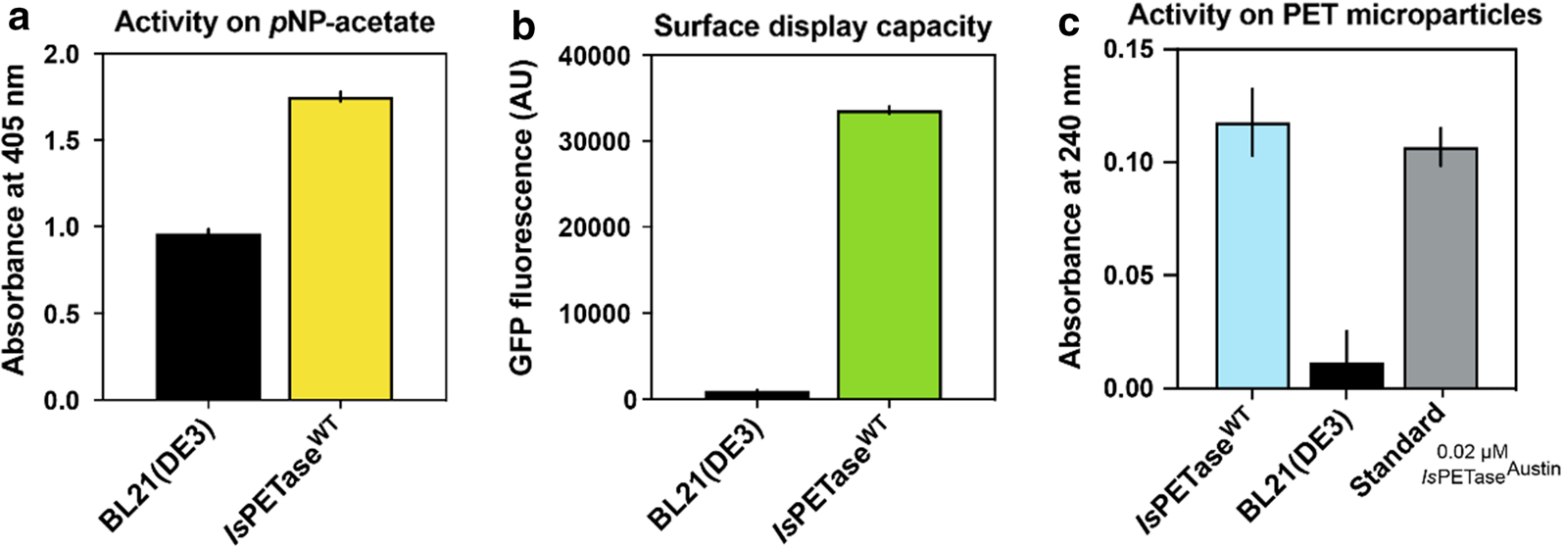
Characterization of surface-bound *Is*PETase^WT^ activity. **a** Activity and **b** whole-cell fluorescence of BL21(DE3) cells expressing *Is*PETase^WT^ within the LppOmpA surface display module compared to BL21(DE3) grown without expression vector. **c** Degradation of PET microparticles (15 g/L) measured via absorbance increase at 240 nm for wt *Is*PETase bound to the bacterial cell surface. Standard: 0.02 µM purified *Is*PETase^Austin^

To eliminate the background activity detected for non-producing *E. coli* cells on *p*NP-acetate, as well as, to investigate whether the surface-bound *Is*PETase protein was folded correctly and in an active state, we next assessed the activity of the *Is*PETase^WT^ towards PET particles. *Is*PETase^WT^ exhibits several fold lower activity towards PET than improved enzyme variants. This makes the wildtype enzyme an ideal candidate to test both baseline sensitivity of the assay and suitability of a surface display strategy for detection of non-optimized PETases. To do so, cell-bound *Is*PETase^WT^ produced in BL21(DE3) harboring expression vector pKSD:LppOmpA-NB*-Is*PETase^wt^ (20 h post induction, expression at 30 °C) was prepared and surface display verified by activity towards *p*NP-acetate (Fig. 3a) and GFP fluorescence (Fig. 3b).

Suspended, semi-crystalline PET powder, was used as substrate and the formation of degradation product was detected via an increase in absorbance at 240 nm. As expected, no shift in absorbance (due to release of PET degradation products) could be detected for non-induced cells not expressing the *Is*PETase surface display module (Fig. 3c). However, significant *Is*PETase activity on free PET could be detected for the surface-displayed *Is*PETase^WT^ while still bound to the cell surface. The activity of the surface displayed PETase corresponded to the activity of 20 nM purified (free) *Is*PETase^Austin^ measured under the same conditions [16] and used here as a positive control. This showed that the *Is*PETase^WT^ was correctly folded and displayed in sufficient quantities for detection.

### Surface display allows expression and activity assessment of different *Is*PETase variants

**Fig. 4:**
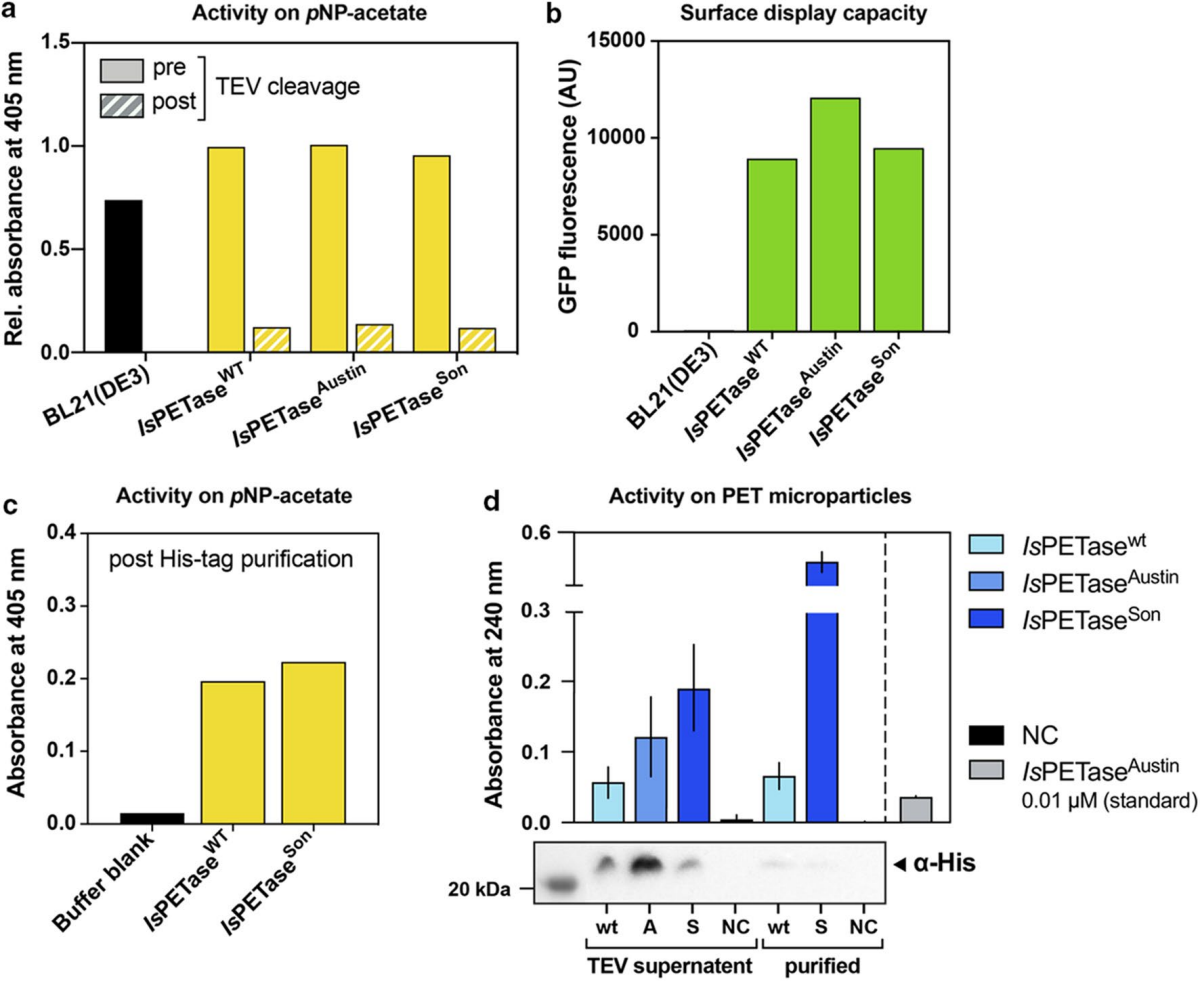
Surface display and activity assessment of different *Is*PETase variants. **a** Activity of wt, Austin, and Son *Is*PETase variants expressed via the LppOmpA surface display module prior to and post TEV cleavage, hence release from the cell surface. To allow comparison of absorbance, values are relative to *Is*PETase^WT^ activity prior to TEV cleavage. Expression of all samples was performed at 16 °C. **b** Whole-cell fluorescence verification of surface-displayed *Is*PETase variants (wt, Austin, Son) prior to TEV cleavage. **c** Activity of *Is*PETase^WT^ and *Is*PETase^Son^ on *p*NP-acetate after His-tag purification. Buffer blank: 50 mM HEPES buffer. **d** Activity and detection of the previously described *Is*PETase variants expressed on the surface and subsequently cleaved off (TEV supernatant) and His-tag purified (purified). Activity of the released *Is*PETase variants on PET microparticles (upper half) and detection of the His-tagged enzymes via western blot (lower half) using an anti-His antibody. NC: Negative control BL21(DE3) cells

Having shown that the *Is*PETase^WT^ was correctly folded when surface-displayed, we next wanted to explore the correlation between the surface-bound *Is*PETase activity measured in the initial screening on *p*NP-acetate with the functionality of the free enzyme on its (actual) substrate PET. This was done by utilizing the TEV cleavage sites present in the anchor module to release all three surface-displayed *Is*PETase variants into the supernatant while removing the insoluble fraction by centrifugation. Measurements of the *p*NP-acetate activity in the soluble fraction clearly showed that all three PETase variants had been successfully released from the cell surface via TEV treatment (Fig. 4a). The background activity previously observed for the BL21(DE3) control strain was now absent, indicating that it could be caused by a native *E. coli* membrane-associated esterase. Solubilization of the *Is*PETase variants by TEV cleavage resulted in an approximate 10-fold loss in absolute activity. This was most likely a combination of inefficient solubilization and, more significantly, removal of the high *E. coli* background activity which accounted for 86–87% of the total activity prior to TEV cleavage (Fig. 4a). TEV-mediated enzyme release thus enables detection of false positive (non-producing or inactive) batches, as it decreases the amount of background activity for the non-expressing *E. coli* production strain significantly. Additionally, the relative abundance of the three *Is*PETase variants either prior or post TEV cleavage was similar, confirming that activity on *p*NP-acetate is not caused by unspecific activity of the *E. coli* background strain but derived from the surface-displayed *Is*PETase variants.

The three *Is*PETase variants chosen for surface display (*Is*PETase^WT^, *Is*PETase^Austin^ and *Is*PETase^Son^) have previously been characterized [7, 9, 11], and therefore, provide an optimal test set of enzymes to verify the screening strategy explored here. While the *Is*PETase^Austin^ variant (S238F and W159H) has improved PET degradation activity due to a narrowed binding cleft [9], Son et al. implemented three point mutations (S121E, D186H and R280A) into the wildtype enzyme increasing its thermal stability (Tm + 8.81 °C), meanwhile, further enhancing the enzyme’s PET degradation activity (14-fold at 40 °C) [7] compared to *Is*PETase^WT^. As the variants have been evolved towards higher specific activity on PET and not *p*NP-acetate, it was expected that *p*NP-acetate is unsuited to resolve clear activity differences (Fig. 4a). Nonetheless, initial activity screening on *p*NP-acetate offers a fast and easy preliminary screening step to select active enzyme variants and reduce sample size for downstream analysis.

We next purified the solubilized *Is*PETase^WT^ and *Is*PETase^Son^ using His-tag purification columns. The two PETase variants were chosen as they both appeared to be surface-displayed at similar levels as determined by GFP fluorescence (Fig. 4b). As a quality control, activity of the purified samples towards *p*NP-acetate was measured. As shown in Fig. 4c, background activity was significantly removed during purification, supporting the earlier notion that this resulted from a native *E. coli* esterase. Even though enzyme amounts post purification were not in sufficient amount to be detected on a SDS-PAGE, all *Is*PETase could be detected using His-tag specific (α-His) antibodies via western blot (Fig. 4d). This was not surprising, as protein surface display suffers from the bottleneck caused by the inefficient and competition-heavy bacterial membrane translocation machinery, as well as the spatial limitation of the cell surface itself [20].

Interestingly, enzyme activity on PET increased after purification even though absolute enzyme amounts decreased, indicating inhibition by other proteins or compounds released during cell lysis present in the unpurified supernatant or better accessibility of the smaller enzyme to the corrugated surface upon removal of potential steric hindrance of the large construct (Fig. 4d). This observation is supported by the presence of multiple proteins visible on the SDS-PAGE of *Is*PETase samples after TEV cleavage but prior to purification (Additional file 1: Figure S2). Activity on PET for the purified *Is*PETase^WT^ enzyme was comparable to its activity in the TEV-cleaved but non-purified sample. In contrast, the purified *Is*PETase^Son^ exhibited a 2.5-fold increase in activity on PET compared to its non-purified sample, and a sevenfold higher activity compared to the purified wildtype enzyme. The latter corresponds well with the increased enzyme activity reported for *Is*PETase^Son^ [7] and supports the hypothesis of optimized substrate binding/enzyme efficiency post purification. Comparing band intensities visible on the western blot (Fig. 4d), the apparent protein concentration of the *Is*PETase^Son^ variant was approximately half of *Is*PETase^WT^. This, combined with the observed 7-fold activity increase on PET, adds up roughly to the 14-fold higher activity previously reported for the variant. Looking at the TEV supernatant of *Is*PETase^Son^ and *Is*PETase^Austin^, a similar comparison of protein concentrations in the western blot and activity towards PET (Fig. 4d) would suggest a lower specific activity of *Is*PETase^Austin^ at the conditions used.

A future refinement would be to include purified samples of selected enzymes, thereby allowing an improved quantification of the total amount of surface displayed enzyme. A potential caveat is that enzymes with large differences in specific activity (as seen for the *Is*PETase variants in this study) could easily bias this analysis. In contrast, using GFP binding as a proxy for quantifying the amount of surface display enzyme functions independently of enzyme specific activity. In summary, we show that an LppOmpA fusion protein can facilitate functional surface display of *Is*PETases and therefore easily be assayed whilst still bound to the cell surface, as well as after release into solution via the simplistic procedure outlined here. Two different surface display modules were investigated in this study, of which the spatial architecture of the LppOmpA module with the C-terminally positioned PETase resulted in the most optimal configuration. Further optimization of the culturing/expression conditions, the TEV cleavage, and the production host strain can be explored for more efficient expression of the *Is*PETase variants in this set-up. Surface display can be used for rapid screening of novel PETase variants enabling a fast and simplistic activity readout on PET. Moreover, the availability of the GFP nanobody allows assessment of expression independently from enzyme activity, which makes it well suited as initial screening tool for new enzyme candidates and allows for easy implementation into high-throughput screening workflows in both academic and industrial settings.

## Supplementary Information

**Additional file 1.** **Figure S1**. Comparison of IsPETase activity on pNP-acetate and -butyrate. Statistical evaluation: Multiple comparison analysis (two-way ANOVA), *: p-value <0.05, ****: p-value <0.0001. **Figure S2**. SDS-PAGE corresponding to western blot in Fig. 4c. S: Protein standard (Novex™ Sharp, ThermoFisher), WT: IsPETase^WT^, A: IsPETase^Austin^, S: IsPETase^Son^, NC^I^: TEV buffer control, NC^II^: BL21(DE3) wt control, NC^III^: HEPES buffer control. **Table S1**. Overview of primers. Primers used to construct vectors pKSD:LppOmpA-NB and pKSD:NB-C-IgAP via uracilexcision (USER) based cloning. **Table S2**. Sequences used in this study. All are based on the IsPETaseWT with the listed mutations in subscript.

## Data Availability

Data sharing is not applicable to this article as no datasets were generated or analysed during the current study. The plasmid vector used for surface display is available from the corresponding author on reasonable request.
