# Supplementary material for: Surface display as a functional screening platform for detecting enzymes active on PET
Source: Microb Cell Fact. 2021 May 1;20:93. doi: 10.1186/s12934-021-01582-7 (PMC8088578; doi:10.1186/s12934-021-01582-7)
Supplement: Supplementary file 1 — Additional file 1. Figure S1. Comparison of IsPETase activity on pNP-acetate and -butyrate. Statistical evaluation: Multiple comparison analysis (two-way ANOVA), *: p-value <0.05, ****: p-value <0.0001. Figure S2. SDS-PAGE corresponding to western blot in Fig. 4c. S: Protein standard (Novex™ Sharp, ThermoFisher), WT: IsPETaseWT, A: IsPETaseAustin, S: IsPETaseSon, NCI: TEV buffer control, NCII: BL21(DE3) wt control, NCIII: HEPES buffer control. Table S1. Overview of primers. Primers used to construct vectors pKSD:LppOmpA-NB and pKSD:NB-C-IgAP via uracilexcision (USER) based cloning. Table S2. Sequences used in this study. All are based on the IsPETaseWT with the listed mutations in subscript. [file 12934_2021_1582_MOESM1_ESM.docx]

**Additional file 1**


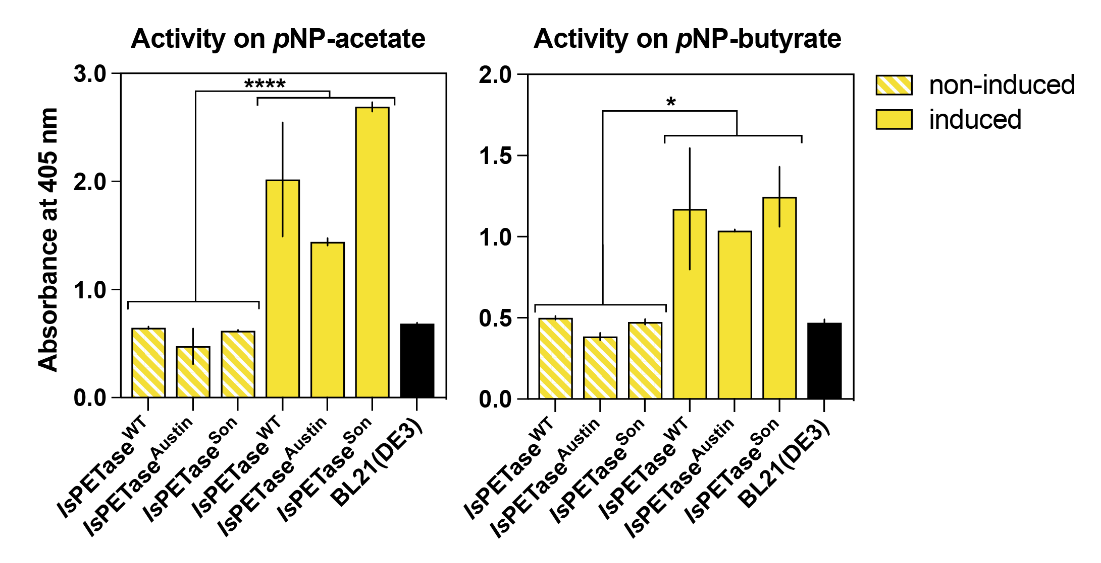


**Figure S1. Comparison of *Is*PETase activity on *p*NP-acetate and -butyrate.** Statistical evaluation: Multiple comparison analysis (two-way ANOVA), *: p-value <0.05, ****: p-value <0.0001.


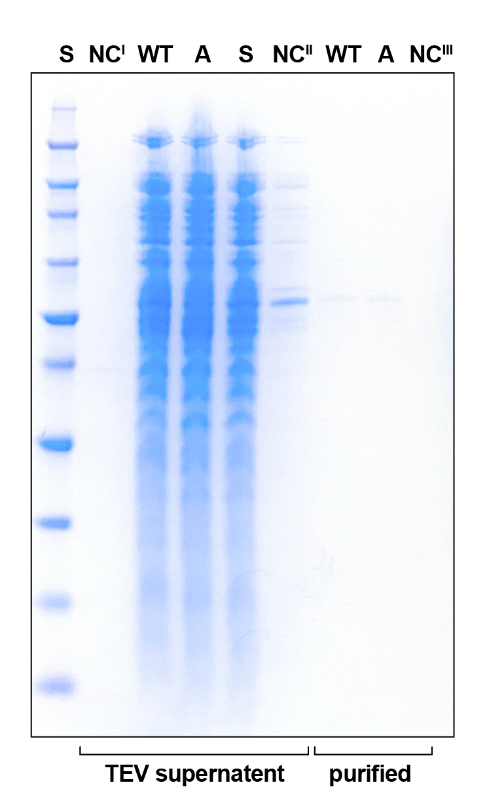


**Figure S2**. **SDS-PAGE corresponding to western blot in Fig**. **4c**. S: Protein standard (Novex™ Sharp, ThermoFisher), WT: *Is*PETase^WT^, A: *Is*PETase^Austin^, S: *Is*PETase^Son^, NC^I^: TEV buffer control, NC^II^: BL21(DE3) wt control, NC^III^: HEPES buffer control.

**Table S1. Overview of primers.** Primers used to construct vectors pKSD:LppOmpA-NB and pKSD:NB-C-IgAP via uracil-excision (USER) based cloning [15].

| Number | Name | Sequence |
| --- | --- | --- |
| 1 | PETase-N-TEV_int_fw | acctgtactUccagggtcagac |
| 2 | PETase-C-STOP_intOmpA_rev | actgcctggacUagtggtggtgatggtggtgc |
| 3 | PETase-C-TEV_intIgAP_rev | atggtggUgctcgagggaac |
| 4 | IgAP_bb_NB-N_fw | accaccaUcaccaccacgaaaacctgtacttccagggtatggctcaggtccaactgg |
| 5 | IgAP_bb_pelB-C_rv | aagtacaggUtttcaattgcggctgaattgtcgatcgg |
| 6 | LppOmpA_bb_NB-C_fw | agtccaggcagUtgaagccaggatagagtcgacctg |
| 7 | LppOmpA_bb_NB-C_rv | aagtacaggUtttcaccaggaccgctggc |

**Table S2. Sequences used in this study.** All are based on the *Is*PETase^WT^ with the listed mutations in subscript

| *Is*PETase^WT^ | gaaaacctgtacttccagggtcagaccaatccgtatgcgcgcggccccaaccctaccgccgcctcgttggaagccagcgcgggaccctttaccgttcgtagctttaccgttagccgtccgtccggatatggtgcagggaccgtctattacccaaccaatgcaggcggcaccgttggcgcgattgcaatcgtccccgggtacaccgcgcgtcaaagcagcattaagtggtggggtccgcgcttagctagccatggctttgtggttattaccatcgatacgaacagcactctagaccagcccagcagccgtagctcgcaacagatggccgcgcttcgtcaagttgcgagcttgaacgggaccagcagtagcccgatttacggaaaggtcgatactgcccgcatgggtgtgatgggctggtcaatggggggcggcggttcacttattagcgccgcgaacaacccgagtttaaaagcagcggcaccgcaggcgccatgggactcttcaaccaacttcagcagtgttaccgtgccgacgctgattttcgcgtgcgagaatgatagcattgcaccggtgaacagcagcgcgctgccgatttatgatagcatgtcccgcaacgcaaaacagtttctggaaattaacggcggtagccactcttgtgccaactctgggaacagcaaccaggcactgatcggaaaaaaaggggttgcatggatgaaacgattcatggataatgacacccgttactcaaccttcgcctgtgagaatcccaacagcacacgcgtgtcggattttcgcaccgcgaactgttccctcgagcaccaccatcaccaccacgaaaacctgtacttccagggt |
| --- | --- |
| *Is*PETase^S238F/W159H^ (Austin *et al*., 2018) | gaaaacctgtacttccagggtcagaccaatccgtatgcgcgcggccccaaccctaccgccgcctcgttggaagccagcgcgggaccctttaccgttcgtagctttaccgttagccgtccgtccggatatggtgcagggaccgtctattacccaaccaatgcaggcggcaccgttggcgcgattgcaatcgtccccgggtacaccgcgcgtcaaagcagcattaagtggtggggtccgcgcttagctagccatggctttgtggttattaccatcgatacgaacagcactctagaccagcccagcagccgtagctcgcaacagatggccgcgcttcgtcaagttgcgagcttgaacgggaccagcagtagcccgatttacggaaaggtcgatactgcccgcatgggtgtgatgggccattcaatggggggcggcggttcacttattagcgccgcgaacaacccgagtttaaaagcagcggcaccgcaggcgccatgggactcttcaaccaacttcagcagtgttaccgtgccgacgctgattttcgcgtgcgagaatgatagcattgcaccggtgaacagcagcgcgctgccgatttatgatagcatgtcccgcaacgcaaaacagtttctggaaattaacggcggtagccacttttgtgccaactctgggaacagcaaccaggcactgatcggaaaaaaaggggttgcatggatgaaacgattcatggataatgacacccgttactcaaccttcgcctgtgagaatcccaacagcacacgcgtgtcggattttcgcaccgcgaactgttccctcgagcaccaccatcaccaccacgaaaacctgtacttccagggt |
| *Is*PETase^S121E/D186H/R280A^ (Son *et al.*, 2019) | gaaaacctgtacttccagggtcagaccaatccgtatgcgcgcggccccaaccctaccgccgcctcgttggaagccagcgcgggaccctttaccgttcgtagctttaccgttagccgtccgtccggatatggtgcagggaccgtctattacccaaccaatgcaggcggcaccgttggcgcgattgcaatcgtccccgggtacaccgcgcgtcaaagcagcattaagtggtggggtccgcgcttagctagccatggctttgtggttattaccatcgatacgaacagcactctagaccagcccgaaagccgtagctcgcaacagatggccgcgcttcgtcaagttgcgagcttgaacgggaccagcagtagcccgatttacggaaaggtcgatactgcccgcatgggtgtgatgggctggtcaatggggggcggcggttcacttattagcgccgcgaacaacccgagtttaaaagcagcggcaccgcaggcgccatggcactcttcaaccaacttcagcagtgttaccgtgccgacgctgattttcgcgtgcgagaatgatagcattgcaccggtgaacagcagcgcgctgccgatttatgatagcatgtcccgcaacgcaaaacagtttctggaaattaacggcggtagccactcttgtgccaactctgggaacagcaaccaggcactgatcggaaaaaaaggggttgcatggatgaaacgattcatggataatgacacccgttactcaaccttcgcctgtgagaatcccaacagcacagcggtgtcggattttcgcaccgcgaactgttccctcgagcaccaccatcaccaccacgaaaacctgtacttccagggt |
